# Physical Activity and Depression in Mothers of a Child With a Special Health Care Need: Informing Future Interventions

**DOI:** 10.1002/puh2.70029

**Published:** 2025-02-07

**Authors:** Brianna J. Black, Tanis J. Walch, Robin L. Dodds, John S. Fitzgerald

**Affiliations:** ^1^ Department of Education, Health and Behavior Studies, College of Education and Human Development University of North Dakota Grand Forks North Dakota USA; ^2^ Division of Special Education & Counseling College of Education, Cal State Los Angeles California USA

**Keywords:** children with special health care needs, depression, mothers, parent support, physical activity, stress and coping

## Abstract

Approximately one in five children in the United States has a special health care need. Mothers of children with special health care needs have worse mental and physical health compared to other mothers. Physical activity (PA) can improve depressive symptoms (DS) and overall health; however, little is known about the acceptability of PA interventions in these mothers. Therefore, the primary aim of this cross‐sectional study was to understand the use of PA in mothers of a child with a special health care need to cope with DS. Specifically, this study examined PA norms, interests, and rationale for participation PA in 348 mothers (age = 39.3 ± 7.3 years; White = 92%; Midwest = 80.1%; employed = 59.2%; prenatal or at birth diagnosis = 51.7%). Most mothers reported meeting PA guidelines before pregnancy but not during pregnancy, postpartum, and currently, and a majority reported elevated DS. Most mothers (85%) of expressed interest in using PA and yoga to cope with DS. The majority of mothers’ viewed PA as a means to increase health and fitness to meet the needs of their children, though they failed to meet the levels of PA associated with DS reduction. Future initiatives should consider population‐specific PA norms, interests, and rationales to increase the salience of meeting PA recommendations in this population.

## Graphical abstract

Mothers of children with special health care needs are more prone to depression and anxiety compared to mothers of typically developing children. Overall, 85% of respondents expressed interest in physical activity as a method for coping. Targeted interventions should accommodate caregiving responsibilities.

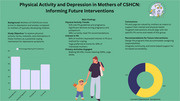

## 1 Introduction

An estimated 13.3 million children, or 18% of all children aged birth to 17 years in the United States, have a special health care need [1]. Special health care needs are defined as chronic physical, developmental, behavioral, cognitive, or emotional disabilities and may occur prior to birth, at birth, or after birth [2]. Following their child's special health care need diagnosis, mothers may experience weight gain, increased chronic disease risk, depressive symptoms (DS), anxiety, and added stressors due to family demands with accommodations [3]. Additionally, mothers of a child with a special health care need (CSHCN) have been shown to have poorer overall well‐being and mental health compared to mothers of typically developing children [4, 5].

Mothers of a CSHCN are more prone to chronic feelings of stress, social isolation, frustration, discrimination, and financial difficulties [6, 7]. Following a child's diagnosis, the mother often has a trauma‐ or grief‐like reaction [8]. Both parents after birth may experience a range of emotions (e.g., shock and anger) that closely resemble posttraumatic stress disorder [8]. Stress and anxiety are the most common health conditions examined amongst women after giving birth to a CSHCN [9]. The stigma of mental health challenges can cause mothers to hide them for fear it will negatively affect their abilities to parent, that they will be reported to child protective agencies, and due to past experiences, a lack of trust in health care providers [8].

Mothers who have a CSHCN may gain additional benefits from engaging in physical activity (PA) and self‐care to reduce mental health symptoms. PA Guidelines for Americans [10] recommend adults engage in at least 150–300 min a week of moderate‐ or 75–150 min of vigorous‐intensity aerobic PA or a combination of moderate‐ and vigorous‐intensity PA for health benefits. Regular moderate‐to‐vigorous PA is associated with reduced anxiety, decreased DS, improved sleep, and improved quality of life in as little as a few weeks or months of participation [10]. In the United States, more than 50% of women do not participate in the recommended amount of PA, and more than 25% of women are completely inactive [3, 11]. Women significantly reduce their activity levels as they transition into pregnancy, postpartum, and motherhood [12]. Research has shown that after having a child, mothers decrease their PA at a rate of 14 h per week, whereas sedentary behaviors and activities such as driving have increased by 6 h [13, 14]. Thus, it is imperative to understand strategies to increase PA in mothers of CSHCNs in order to embed a protective factor to support their mental health.

Evidence suggests that maintaining higher levels of PA during and after the transition into motherhood may be protective against depressive and anxiety symptoms. Higher levels of moderate PA (i.e., biking, house cleaning) in women during life events, particularly with aging, marriage, and having a baby have been shown to be associated with lower DS [15]. Regular PA (e.g., aerobics and/or strength training) three to five times per week for 30–60 min can provide a decrease in depressive symptomology, and an increase in overall health benefits [16]. Furthermore, PA during and after pregnancy is associated with fewer DS compared to inactive pregnant and postpartum women [17]. Yoga has been perceived as a stress management tool that can assist in alleviating depression and anxiety disorders. Practicing yoga can be considered a supplementary treatment option for all patients with depressive disorders and individuals with elevated levels of depression [18]. Yoga has also been shown to increase PA and help with mental health in mothers after a traumatic event, such as stillbirth [19], as well as improve sleep quality and reduce stress in mothers of children with intellectual and developmental disabilities [20].

Mothers of a CSHCN are more willing to seek support for their children than for themselves [21] and need strategies to improve mental health and decrease DS. Positive coping strategies that focus on the mother, such as PA (e.g., yoga, walking), online or peer support, or spending time by themselves, may reduce stress levels and help aid in self‐care [22, 23]. The prevalence of moms of a CSHCN meeting PA guidelines is unknown but likely low. Given the low prevalence of those meeting PA guidelines, PA interventions aimed at increasing PA have the potential to achieve substantial reductions in depressive and anxiety symptoms at the subpopulation level. Unfortunately, a gap exists regarding the type of PA interventions that are acceptable for mothers of a CSHCN. Further understanding of PA norms in mothers of a CSHCN would better clarify the need for population‐specific interventions and improve the ability of practitioners and researchers to prescribe PA in line with population‐specific PA norms, which may increase long‐term adherence. Thus, the primary aim of this study was to examine PA norms, interests, and rationale for participating in PA in mothers who have a CSHCN to improve DS and inform future public health interventions.

## 2 Methods

### 2.1 Participants and Procedures

American and Canadian women were eligible to participate in the study if they were the primary caregivers of a child diagnosed (birth through 17 years of age) with a special health care need or disability. Women who were currently pregnant, who had experienced a stillbirth, or loss of a child were excluded from this study.

This was a cross‐sectional, descriptive exploratory study and was approved by the Institutional Review Board at the University of North Dakota. Participants were recruited locally and nationally through collaboration with advocacy organizations, email list‐serves, and social media (e.g., Facebook). Participants were recruited electronically from 28 February 2020 to 10 March 2020. Participants provided informed consent prior to the start of the survey and were provided a $10 gift card for their voluntary participation.

### 2.2 Measurement

The survey included the following four sections: (1) general information of child and child's diagnosis (seven items). Questions in this section were related to the child's age, description of diagnosis, child's age at time of diagnosis, and whether diagnosis was prenatal. (2) PA over time included PA they engaged in before pregnancy, during pregnancy, during the postpartum period, and currently (16 items). (3) Depressive Symptomology (10 items) related to parenting a CSHCN. (4) Demographic Characteristics (17 items) included relationship to child, race, ethnicity, and income. The average time to complete the survey was 12 min.

#### 2.2.1 General Information of Child and Child Diagnosis

Participants were asked to provide general information about their child and their child's diagnosis. This included complications during pregnancy and prenatal or postnatal diagnosis.

#### 2.2.2 PA Participation and Health of Mother

A modified version of the PA of Change Questionnaire [24] was used to assess PA during four points of time (before pregnancy, during pregnancy, postpartum period, and currently). Participants were provided a description of PA [10] and then asked two questions regarding their PA at each of the four time points. An example question includes, “In the time since your child was born, have you participated in exercise or PA?” Skip logic approaches were used to examine participants engagement of PA and coping with DS, anxiety, and/or feelings associated with their child's special health care need [24].

Next, participants were asked whether they were interested in engaging in PA as a means to cope with DS, anxiety, and/or grief associated with the diagnosis of their child (Yes or No response). The same question was also asked for yoga (Yes or No) and if they had ever tried to use yoga as a means to cope (Yes or No). If they answered yes to being interested in using yoga as a means to cope with DS, they ranked the settings (1 = most preferred to 5 = least preferred) they preferred to participate in yoga (e.g., in your own home, yoga studio, group‐based setting at hospital/doctor's office, gym, other). If they selected Other, they could write in the setting they preferred [24].

#### 2.2.3 Depression and Emotional Health

A modified Edinburgh Postnatal Depression Scale (EPDS) was used to determine depressive symptomatology associated with parenting a CSHCN (*α* = 0.89). The 10‐question EPDS included 10 statements that described feelings reflective of depression symptoms during the last 7 days on a scale from 0 (never) to 3 (quite often). The clinical threshold of 10 on a 30‐point scale was scored as possible depression. The EPDS is a consistent tool for women who have experienced extreme emotional duress after having a child [25].

#### 2.2.4 Mother and Child Demographics Information

Participants self‐reported (17 items) demographic characteristics, such as relation to the child, age, state and municipality area they currently reside in (e.g., rural, metropolitan), community reside in (American Indian Reservation), race, ethnicity, employment status, and annual income.

### 2.3 Data Analysis

Descriptive statistics (e.g., means, percentages) were calculated for all survey questions using SPSS (v26). Qualitative responses were summed and categorized to understand their interest in the type of PA they engaged in and preferred.

## 3 Results

Mothers (*n* = 348) were an average age of 39.3 years (SD = 7.3), primarily Caucasian (92%), non‐Hispanic (95.1%), and lived in the Midwest (80.2%). Almost 40% of the sample lived in North Dakota (39.1%), in a metropolitan area (45.1%), and were employed for wages (59.2%). Approximately one‐third of mothers (33.1%) received their child's diagnosis within the last 3 years, and over 50% of mothers received a prenatal or at birth diagnosis. The average child age at diagnosis was 1.7 years (SD = 3.0), with the average age of the child currently was 8.3 years (SD = 4.9). Participant demographic characteristics are reported in Table 1.

**TABLE 1:** Participant demographic characteristics (*n* = 348).

Mother's age, years (SD)	39.3 (7.3)	
	N	%
**Biological mother**	329	94.5
**Adoptive mother**	19	5.5
**Race**		
Caucasian or White	320	92.0
African American or Black	8	2.3
Asian Pacific Islander	6	1.7
American Indian or Alaskan Native	8	2.3
Other	5	1.4
No answer	1	0.3
**Ethnicity**		
Non‐Hispanic	331	95.1
Hispanic	17	4.9
**Geographic region**		
Out of United States	1	0.3
Northeast	6	1.7
Midwest	279	80.2
South	32	9.2
West	30	8.6
**State of North Dakota**		
North Dakota	136	39.1
Other state	212	60.9
**Population density area**		
Rural (<2500)	40	11.5
Large rural (2500–9999)	54	15.5
Micropolitan (10,000–49,999)	95	27.3
Metropolitan (>50,000)	157	45.1
No answer	2	0.6
**Live on American Indian reservation**		
Yes	6	1.7
No	339	97.4
No answer	3	0.9
**Employment**		
Employed for Wages	206	59.2
Self‐employed	22	6.3
Out of work <1 year	6	1.7
Out of work >1 year	11	3.2
Homemaker	85	24.4
Student	7	2.0
Retired	1	0.3
Unable to work	10	2.9
**Income**		
<Than $20,000	65	18.7
$20,000–$39,999	67	19.3
$40,000–$59,999	73	21.0
$60,000–$74,999	44	12.6
$75,000 or more	92	26.4
Missing data	7	2.0
**Prenatal or at birth diagnosis**	180	51.7
**Child age at time of diagnosis, year (SD)**	1.69 (3.0)	
**Age of child now, year (SD)**	8.3 (4.9)	
**Time since child diagnosis, year (SD)**	6.4 (4.5)	
Less than 1 year	16	4.6
1–3 years	99	28.5
4–6 years	82	23.6
7–9 years	63	18.1
10 years or more	88	25.2

### 3.1 PA Participation

Mothers reported meeting PA guidelines before pregnancy (51%), during pregnancy (31%), postpartum (within 1 year; 30%), and current participation (39%). A majority (59.8%) of mothers reported elevated DS. Most mothers reported engaging/talking with friends and family (74%), participating in PA (37%), taking anti‐depressive medication (35%), utilizing a support group (28%), or other (18%) as a means to cope with their DS. Table 2 reports qualitative responses to “other” ways they cope with depression, anxiety, and feeling associated with their child's special health care need. Over 77% (*n* = 268) of mothers participated in some type of PA and did so to help with DS (37%), better overall health (76%), better quality of life (77%), and weight loss (57%). Mothers who engaged in PA (37%) primarily used walking (65.6%), house cleaning (56%), yoga (40%), strength training (38%), jogging (33%), and “other activities” (23%) to cope with depression. Table 3 reports the qualitative responses to “other forms of PA” that participants are currently engaged in. Table 4 reports the qualitative responses for other reasons why they participate in PA, which included: cannot die due to my son's needs, have to remain fit/strong to physically care for my child, to be strong enough to care for my daughter, self‐love, and for personal time away. Interestingly, 85% of all participating mothers were interested in using PA (85%) and yoga (71%) as a means to cope with DS. Approximately one‐third (36%) of participants had tried yoga as a means to cope with DS. Of those interested in using yoga, almost half (45%) preferred to participate from their home. In addition, nearly one third of respondents preferred using a studio or gym (36% and 34%), respectively.

**TABLE 2:** Qualitative responses to “other” ways mother's cope with depression, anxiety, and feelings associated with their child's special health care need, *n* = 63 (18.1%).

Advocacy (*n* = 1)	Nothing, wait for it to pass (*n* = 3)
Alone time (*n* = 5)	Plans to join a support group (*n* = 1)
Art, crafts, quilt, sewing (*n* = 7)	Prayer and/or church (*n* = 9)
Television (*n* = 1)	Reading (*n* = 7)
Chocolate (*n* = 2)	Relaxing (*n* = 1)
Cleaning (*n* = 2)	Take a long shower or hot tub (*n* = 2)
Crying (*n* = 2)	Self‐care (*n* = 1)
Facebook groups (*n* = 1)	Shopping alone (*n* = 1)
Gaming (*n* = 1)	Sleep (*n* = 2)
Hobbies (*n* = 4)	Spousal support (*n* = 2)
I don't (*n* = 8)	Work (*n* = 2)
Music (*n* = 2)	Yoga (*n* = 1)

**TABLE 3:** Qualitative responses to other physical activities engaged in to cope with depressive symptoms, *n* = 29 (22.7%).

Barre classes (*n* = 1)	Horseback riding (*n* = 1)
Crossfit or high intensity interval training (*n* = 7)	Pilates
Cycling or group exercise classes (*n* = 2)	Playing and/or running with my kids
Dancing (*n* = 2)	Swimming (*n* = 5)
Elliptical (*n* = 3)	Tai chi (*n* = 1)
Hiking (*n* = 2)	Zumba (*n* = 2)

**TABLE 4:** Qualitative responses to other reasons to participate in physical activity, *n* = 28 (10.4%).

Art	Better interaction with my kids
Cannot die due to son's needs	Doctor recommended
Energy	Family time
Focus on me	For personal time away
I don't have time to do that, but I do some activity with my job and housework	Have to remain fit/strong to physically care for my child
General health	I have to
I walk a lot at work, and quite fast and frequently (I don't exercise outside of work)	To accomplish what the task is (e.g., gardening, travelling to a destination by booking/walking)
Injury prevention	Time to myself
Mental focus	My children are young, and I am almost 50
Needing a break while walking the dog	Respite
Self‐love	Spending time being active with my children
To be strong enough to care for my daughter	To do something for myself
To be strong to manage my child physically as needed for his safety	

## 4 Conclusions

To our knowledge, this was the first study to examine the acceptability, preferences, and experiences with PA for mothers of a CSHCN. As such, this research offers insight for health care providers to design and implement interventions at the time of their child's diagnosis that includes PA and/or yoga to improve mental and physical health in mothers of a CSHCN. Thus, the primary aim of this study was to examine PA norms, interests, and rationale for participating in PA in mothers who have a CSHCN to improve DS and inform future public health interventions.

Across the country, almost 20% of families are raising a CSHCN and function differently from other families. Mothers are typically the primary caregiver and have the most knowledge regarding the child's health, development, service needs and interactions, and experience higher rates of unrelenting stress and adverse mental health issues [26]. Although the mother is often the primary caregiver, special health care diagnosis affects the entire family, as routines may be based around the child's needs and the family may sacrifice time together, family activities, and their own health for requirements of their child's care [27, 28, 29].

Mothers who have a CSHCN are at greater risk for depression, anxiety and stress [30]. Mothers who have a child with autism spectrum disorder (ASD) report higher levels of DS and fatigue, both stemming from poor quality of sleep, lack of PA, and increased need for social support [31]. Additionally, Roberts and colleagues [32] report that maternal posttraumatic stress disorder may be associated with their child's ASD diagnosis. This may be from having maternal stress and due to PTSD and ASD sharing the same genetic risk [32]. Results from our study suggests that mothers of a CSHCN are interested in utilizing PA and yoga to cope with DS. Thus, it is critical that health care providers and support programs for mothers caring for a CSHCN incorporate PA into their family‐centric strategies to improve both physical and mental health.

A decrease of PA from before pregnancy to postpartum was noted. Such reduction can be due to demands of caring for a child who has a special health care need, overall demands of being a mother, and physiological changes to the body [33]. Women frequently gain more weight than needed during pregnancy, and then fail to lose the weight by the 6‐month postpartum period [34]. In addition, pre‐pregnancy weight has also been shown to contribute to long‐term obesity, as women who start pregnancy heavier retain more weight following childbirth [35]. The slight PA increase from the postpartum period to currently in this study may be due to the way it makes a woman feel by working out (i.e., strong, empowered), disease/obesity prevention, and women having time to take care of themselves (self‐care). After a traumatic birth complication or their child's special health care diagnosis, women may use PA to cope, have time alone, and recuperate [36]. As physiological changes happen to a woman to meet the demands of being a mother, moderate‐to‐vigorous PA (e.g., walking, biking, and yoga) can act as a protective, non‐pharmacological method to decrease depressive and other mental health symptoms [37]. Long‐term strategies are needed to encourage women to stay active while having a CSHCN. In particular, health care providers are in an optimal position to intervene at time of diagnosis, as helping the mother have optimal physical and mental health will also benefit the child. This is particularly important, as leisure‐time PA before, during, and after pregnancy may reduce the risk of postpartum depression [38].

Increased positive health behaviors, such as PA, are associated with better mental health outcomes. Results from our study showed that over half of the mothers (59%) exhibited elevated DS, and approximately one‐third (39%) currently participate in the recommended amount of moderate‐to‐vigorous PA. PA has been shown to reduce stress, decrease DS, and improve obesity and overall fitness in women [39]. Teychenne and colleagues [40] examined over 1500 adult women and found that at least 3.5 h per week of leisure‐time PA had lower DS and lower odds of DS. Adding or increasing routine bouts of PA, especially during a challenging time in their lives (i.e., marriage, birth, and new job) has been found to be positively associated with mental health [41, 42]. Specifically, Brown and Trost [41] found that women ages 18–23 years of age were more likely to become inactive after getting married, having a child, or beginning a new job. Thus, maternal PA interventions are needed to improve health, but more importantly, that are appropriate and reasonable in this underserved population of mothers. Mothers of a CSHCN have unique barriers to PA, so understanding what is feasible is of critical public health importance.

In our study, over one‐third of participants reported the use of PA as a means to cope with DS. Others reported engaging with family/friends and using anti‐depressive medications to cope with their DS. Adapted from an ancient holistic health system, yoga is a newer mainstream practice in the United States, especially as use for coping with mental health symptoms. Yoga is linked to health promotion, gentle PA, and coping with stressors and disease [43]. In our study, over two‐thirds of women were interested in using yoga as a coping mechanism for DS, anxiety, and grief. On the basis of research on pregnant women and yoga as a healing practice, this may be another opportunity to increase maternal PA following their child's diagnosis.

Women, specifically mothers, are less likely to engage in forms of PA compared to men due to barriers they may encounter when having a child, such as lack of time due to caring for a child, guilt associated with being away from the child, and lack of energy [44]. In our study, walking was the highest ranked form of PA by mothers. Walking is often used in health promotion for PA, as it is inexpensive, little to no equipment is needed, which is an activity mothers can accommodate around the family [45]. One strategy that may help improve maternal physical and mental health may be family‐centric strategies, such as walking together. Walking regularly is also a good predictor of lowering rates of chronic diseases in women. This in return provides a better quality of life and helps provide better overall physical health, which is what participants reported in our study [46]. In our study, mothers reported other reasons to participate in PA, such as avoiding injury, self‐care, and maintaining a level of strength appropriate to care for their child. Mothers also reported participating in yoga in their own home as the strongest preference. Understanding these PA norms in mothers of a CSHCN provides important insight to increasing PA in mothers to help reduce anxiety and depression, as well as improve their ability to care for their CSHCN.

To our knowledge, this was the first study to examine PA in mothers caring for a CSHCN and has several strengths and limitations. Strengths included the understudied population included in this study of mothers who have a CSHCN (20% of all families in the United States). Second, this study focused on maternal health, whereas most research in families with a CSHCN are focused on the child [47, 48]. Last, a large sample size of mothers participated in this study. There are also several limitations to note. First, the sample was 92% Caucasian, most were employed, and almost half lived in a metropolitan area. Thus, our means to understand underserved communities and populations was limited. Second, PA was self‐reported by mothers, and they did not report amount of time (in minutes) of PA achieved per week. It is well known that self‐reported PA can be overestimated, and PA recommendations may not have actually been met [49]. Moreover, participants were not asked about other health conditions or weight status, which may impact their ability to participate in PA in which we cannot rule out reverse causality.

The quantitative and qualitative data suggest the majority of mothers’ view PA as a means to increase health and fitness to meet the needs of their children with a special health care need, though fail to meet the levels of PA associated with DS reduction. Future interventions and public health initiatives should consider population‐specific PA norms, PA interests and PA rationale to increase the salience of meeting PA recommendations in this vulnerable population.

## Author Contributions

**Brianna Black:** conceptualization (lead), writing – original draft (lead), formal analysis (lead), writing – review and editing (equal). **Robin Dodds:** review and editing (equal). **John Fitzgerald:** review and editing (equal). **Tanis Walch:** supervision, funding acquisistion, conceptualization (supporting), writing – original draft (supporting), writing – review and editing (equal).

## Ethics Statement

This study and was approved by the Institutional Review Board at the University of North Dakota prior to data collection.

## Consent

All participants were required to read an informational cover letter prior to online participation to ensure informed consent.

## Conflicts of Interest

Dr. Tanis J. Walch received a mini‐grant research award from the College of Education and Human Development for participant incentives. Dr. Tanis J. Walch was a Family Voices of North Dakota Board Member during the data collection phase of this study. This study was completed as a Master's Thesis by the first author.

## Data Availability

The datasets generated during this study are available from the corresponding author on a reasonable request.
